# Role of polyphenols in remodeling the host gut microbiota in polycystic ovary syndrome

**DOI:** 10.1186/s13048-024-01354-y

**Published:** 2024-03-27

**Authors:** Ping Zhou, Penghui Feng, Baoying Liao, Lin Fu, Hongying Shan, Canhui Cao, Renxin Luo, Tianliu Peng, Fenting Liu, Rong Li

**Affiliations:** 1 Center for Reproductive Medicine, Department of Obstetrics and Gynecology, Peking University Third Hospital, No. 49 HuaYuan North Road, Haidian District, Beijing, 100191 China; 2 Key Laboratory of Assisted Reproduction, Ministry of Education, Peking University, Beijing, China; 3 Beijing Key Laboratory of Reproductive Endocrinology and Assisted Reproductive Technology, Beijing, China; 4 National Clinical Research Center for Obstetrics and Gynecology, Peking University Third Hospital, Beijing, China; 5 Department of Obstetrics and Gynecology, Peking Union Medical College Hospital, National Clinical Research Center for Obstetric & Gynecologic Diseases, Chinese Academy of Medical Sciences & Peking Union Medical College, Beijing, China

**Keywords:** Polycystic ovary syndrome, Gut microbiota, Polyphenols, Anthocyanins, Green tea catechins

## Abstract

Polycystic ovary syndrome (PCOS) is a common reproductive and metabolic condition in women of childbearing age and a major cause of anovulatory infertility. The pathophysiology of PCOS is complex. Recent studies have reported that apart from hyperandrogenism, insulin resistance, systemic chronic inflammation, and ovarian dysfunction, gut microbiota dysbiosis is also involved in PCOS development and may aggravate inflammation and metabolic dysfunction, forming a vicious cycle. As naturally occurring plant secondary metabolites, polyphenols have been demonstrated to have anticancer, antibacterial, vasodilator, and analgesic properties, mechanistically creating putative bioactive, low-molecular-weight metabolites in the human gut. Here, we summarize the role of gut microbiota dysbiosis in the development of PCOS and demonstrate the ability of different polyphenols - including anthocyanin, catechins, and resveratrol - to regulate gut microbes and alleviate chronic inflammation, thus providing new insights that may assist in the development of novel therapeutic strategies to treat women with PCOS.

## Introduction

Polycystic ovary syndrome (PCOS) is a commonly occurring, highly heterogeneous endocrine condition in women of reproductive age and a major cause of anovulatory infertility [1]. Its clinical manifestations mostly include oligo-ovulation or anovulation, insulin resistance (IR), excessive androgen secretion, and ovarian polycystic changes, leading to menstrual cycle irregularity and infertility symptoms. Epidemiological research results have shown that PCOS is a serious gynecological condition with high incidence rates in China, the United States, India, and European countries like Spain and Denmark [2, 3]. Women with PCOS have a higher risk of obesity, hypertension, metabolic syndrome, and cardiovascular disease. Moreover, the condition causes fertility difficulties to arise, which negatively impacts the physical and mental health of affected patients. Therefore, the pathogenesis of the condition requires elucidation to develop new approaches to treating PCOS patients and improve their quality of life.

The etiology of PCOS is complex, its clinical manifestations are heterogeneous, and its pathogenesis is yet undetermined. The relationship between the gut flora and PCOS has been extensively investigated in recent years. Studies have analyzed women with PCOS [4–6] and animal models of PCOS [4, 7, 8] to report that there exists a significant difference in the composition of intestinal flora between those with PCOS versus normal controls. This difference is manifested by decreased beta diversity and an altered abundance of specific genera, suggesting a possible correlation between intestinal flora and the development of PCOS [5, 6]. Moreover, following more extensive investigations, the pathological mechanism underlying PCOS has been expanded to no longer be limited to the dysfunction of the hypothalamic-pituitary-ovarian axis, as considerable attention has been redirected toward immune factors. The current study considers the immunological mechanism involved in PCOS and focuses on the dysfunction of immune cells and the abnormal expression of inflammatory factors [9, 10].

**Fig. 1:**
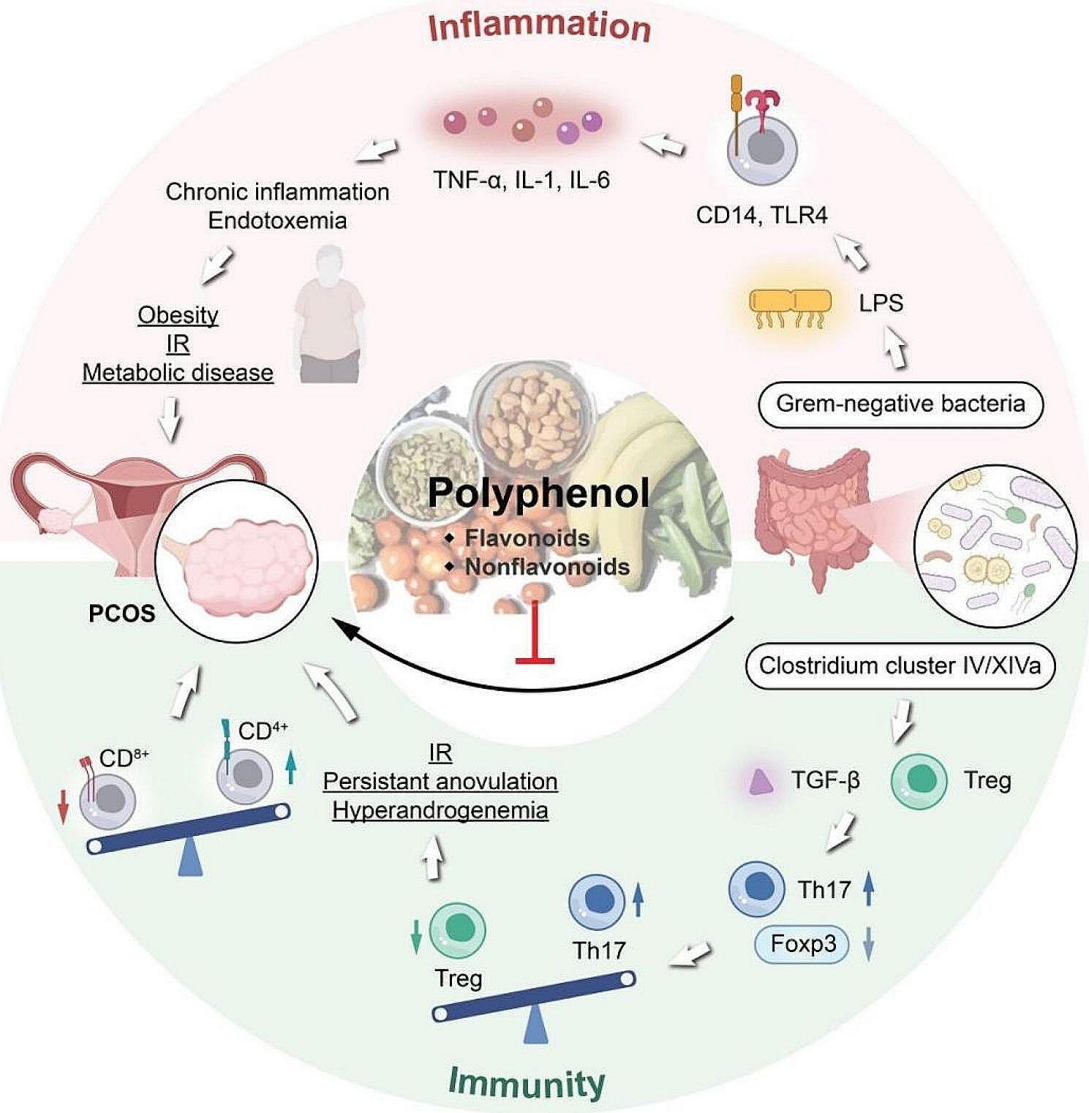
Relationship between polycystic ovary syndrome and the gut flora

Polyphenols are naturally occurring plant secondary metabolites that have multiple phenol units (characterized by having several hydroxyl groups on aromatic rings) and include four principal classes: phenolic acids, flavonoids, stilbenes, and lignans. Secondary metabolites with polyphenol structures are widely found in tea, grapes, apples, alcohol, and various fruits and vegetables. They may have a therapeutic role in alleviating a range of chronic conditions, such as cardiovascular diseases, hypertension, obesity, and neurodegenerative diseases [11]. Studies have shown that dietary polyphenols can exert positive prebiotic effects by promoting the development of beneficial bacteria (i.e., *Lactobacillus* and *Bifidobacterium*), thus maintaining gut microbial balance and supporting gut health by inhibiting pathogenic bacteria [12]. In the current study, we review the role of the gut microbiota in the occurrence and development of PCOS. We also determine the effects of polyphenols on the regulation of gut microbial communities and the inflammatory response to PCOS, indicating the potential of these compounds in the prevention and treatment of the condition (Fig. 1).

## Overview of polycystic ovary syndrome

PCOS is a highly heterogeneous endocrine and reproductive disorder affecting 5-20% women of reproductive age [13]. Due to the complicated clinical manifestations of PCOS, the therapeutic strategies are usually anti-symptomatic and not precise, which increases the treatment cost and brings heavy burden on PCOS patients and society. PCOS has complex etiology. According to the current studies, Dumeric et al. investigated PCOS etiology through evolutionary perspectives, indicating that ancient fat storage mode may contribute to metabolic disorders in PCOS and impairs the long-term health [14]. Besides, genetic factors and maternal influence play important roles in PCOS development. It’s reported that the incidence of PCOS in monozygotic twin sisters was two times higher than that in dizygotic twin and other sisters [15]. Families studies revealed higher prevalence in the first-degree family relatives of PCOS patients [16], indicating the existence of genetic basis in PCOS. Recent years, genome-wide association studies (GWAS) and clinical studies clarified that genes related to endocrine disturbance (*DENND1A* for androgen excess and *FSHR* for low FSH levels) [17], metabolic disorders (RAB5B for glucose metabolism dysfunction) [18] and anovulation (*LHCGR* and *INSR*) [19], which provided profound and specific understanding of genes responsible for PCOS [20, 21].

Environmental factors also contribute to the development of PCOS. Endocrine-disrupting chemicals (EDC) that exist ubiquitously in the environment (such as bisphenol A (BPA), which is derived from disposable plastic cups) enter the body directly or indirectly to consequently affect hormone metabolism in humans [22]. These chemicals can disrupt estrogen balance, which may be a risk factor for the development of PCOS. It has been suggested that BPA is related to the pathogenesis of PCOS [23]. BPA has weak estrogenic effects and can increase the secretion of ovarian androgens in most animals [24]. After BPA accumulation has occurred in the body of patients with PCOS, it has also been found to delay and reduce the clearance of excess androgens in the body, thereby aggravating metabolic dysfunction and increasing the risk of PCOS [25]. Hyperandrogenemia is an essential endocrine feature of PCOS, and the androgen-excessive environment can inhibit follicle development and maturation, resulting in follicular atresia and decreased ovarian estradiol levels [26]. Obesity, IR, and hyperinsulinemia are also major features of PCOS, which modulate ovarian function by interacting with gonadotropin. An elevated level of gonadotropin can amplify luteinizing hormone-induced ovarian androgen production and ultimately prevent ovulation [27].

Clinical studies have shown that patients with PCOS generally exhibit chronic low-grade inflammation, which can also promote the occurrence and development of PCOS [28]. It has been observed that a high degree of macrophage and lymphocyte infiltration occurs in ovarian tissue biopsies obtained from patients with PCOS, and it is presumed that these inflammatory responses can cause insulin resistance and hyperandrogenemia [29]. Moreover, inflammatory cells in the serum are inhibited and reduced after using anti-hyperglycemic agents, adding further support that PCOS is associated with the inflammatory response [29].

## Relationship between polycystic ovary syndrome and the gut flora

### Overview of the gut flora

The intestinal tract comprises the largest area of the body that comes into contact with the external environment and harbors the greatest number of intestinal flora. More than 1,000 kinds of bacteria are colonized in the human gut, and the total amount of bacterial cells contained within is 1.3 times the total number of human cells [30], rendering it known as the “human second genome.” The mammalian gut microbiota is mainly derived from nine phyla, 90% of which belong to two categories: gram-positive Firmicutes and gram-negative *Bacteroides*, followed by Actinobacteria, Proteobacteria, *Clostridium*, and so on. Regarding the physiological characteristics of the gut microbiota, it has been reported that the number of anaerobic bacteria comprising the gut microbiota is two to three orders of magnitude greater in normal human gut flora when compared to that of facultative anaerobic and aerobic bacteria, which can be classified into primary and secondary flora according to colonizing features [31]. The number of major bacteria groups resides in the range of 10^7^-10^8^ cells /g and above, which are mainly comprised of native or resident bacteria, including *Eschobacterium*, *Bacteroides*, *Bifidobacterium*, and others. Secondary flora is classified as those in quantities of 10^7^-10^8^ cells /g and below, which are mainly foreign or passing bacteria, including *Streptococcus*, *Enterococcus*, *E. coli*, and *Lactobacillus* [32]. In terms of their overall effect on human health, the gut flora can be divided into those that are beneficial, intermediate, and harmful, depending on their relationship with the host. Beneficial bacteria include *Lactobacillus*, *Bifidobacterium*, *Enterococcus*, *Lactococcus*, and *Pedioccocus*, which can inhibit harmful microorganisms, enhance host immunity, promote nutrient absorption, synthesize vitamins, reduce cancer occurrence, counteract infection, decompose harmful substances, promote discharge, and relieve allergic reactions. Deleterious bacteria include *Staphylococcus*, *Salmonella*, and *Campylobacter*, which can produce toxins and increase the incidence of cancer, leading to various disorders and infections of the intestinal system. Having normal and symbiotic intestinal flora is of considerable importance to maintaining human health, as it can affect the proliferation of host cells, vascular formation, intestinal endocrine function, nerve signaling, and so on. Thus, an imbalance in the intestinal flora can cause a wide range of systemic diseases, including local gastrointestinal diseases, neurological diseases, respiratory diseases, metabolic diseases, liver diseases, and cardiovascular diseases [33–35].

### The correlation of gut flora composition with PCOS

PCOS presents a correlation between its pathological processes and the alteration of intestinal flora. The study of this correlation is presently focused on metabolic features, including hyperandrogenemia, IR, and metabolic syndrome [36, 37]. The dysbiosis of gut microbiota (DOGMA) hypothesis to explain the underlying cause of PCOS pathogenesis was proposed in 2012 by Tremellen et al [38]. The hypothesis suggests that gut microbiota dysbiosis impaired the intestinal mucosal barrier and elevated intestinal mucosal permeability, which promoted the passage of lipopolysaccharides (LPS) from intestinal to systemic circulation, thus contributing to the progress of hyperandrogenism, anovulation and insulin resistance. Consequently, a greater amount of androgen is produced, and normal follicle development is disrupted. Furthermore, mounting evidence indicates that gut microbiota dysbiosis is characteristic of women with PCOS. Torres et al. observed lower alpha diversity in PCOS patients compared to healthy women and found that hyperandrogenism, total testosterone, and hirsutism were negatively correlated with α diversity, indicating the possible role of the gut microbiota in modulating steroid hormone synthesis [4]. In addition, it was reported that the relative abundance of bacteria from the phylum Tenericutes and phylum Bacteroidetes was significantly lower in PCOS women [39]. Qi et al. analyzed gut flora in patients with PCOS, which appeared to form two different clusters when compared between PCOS and healthy control samples, exhibiting a higher degree of similarity between samples of the same group [5]. Among all the differential species, *Bacteroides vulgatus* (*B. vulgatus*) was reported to contribute the most to the disparity, with its abundance significantly greater in patients with PCOS. Between the PCOS and control groups, functional analysis indicated that the biosynthesis of steroid hormones and secondary bile acids was particularly different between them. Fecal and serum metabolomic analysis revealed significantly lower amounts of both glycine deoxycholic acid (GDCA) and tauroursodeoxycholic acid (TUDCA) in the PCOS group, which may be associated with an increased abundance of bile salt hydrolase. Mechanistically, *B. vulgatus* is involved in bile acid metabolism and leads to decreased levels of GDCA and TUDCA in women with PCOS, which inhibits the secretion of interleukin 22 from intestinal innate lymphoid cell 3 (ILC3), thus contributing to the development of ovarian dysfunction and metabolic disorders in PCOS [5, 6].

### The correlation of the gut flora with PCOS immunity

Intestinal microbes play an important role in the pathological development of various autoimmune and chronic immune diseases, even to the extent to which they are directly related to their disease mechanisms. Intestinal flora in the gut promotes the development and maturation of the intestinal mucosal immune system. When intestinal microorganisms resist pathogen invasion, they can stimulate humoral immunity throughout the intestinal mucosa and immediately produce tolerance. The gut flora balances the systemic immune response by inducing systemic chronic inflammatory responses, regulating T lymphocyte differentiation, encoding immunoglobulin, promoting B cell maturation, and stimulating the production of B cells with antibodies [40]. T lymphocytes primarily function to maintain immune homeostasis. They can be classified into helper T cells (Th), cytotoxic T cells (CTL or TC), regulatory T cells (Treg), and CD4^+^ or CD8^+^ cells, depending on the immune effector functions of the T lymphocytes. Th1/Th2 balance is the main mechanism underlying the balance and maintenance of bodily immunity. Lang et al [41] showed that in the peripheral blood of patients with infertility resulting from PCOS, the Th1/cytokine ratio and Th1/Th2 ratio were higher than normal. Moreover, Th1 and Th2 imbalance leads to poor egg quality, aberrant ovulation, and a reduced pregnancy rate in patients with PCOS, increasing the risk of abortion. These findings suggest that the immunological characteristics of patients with PCOS are mainly defined by Th1-dominated cellular immunity. Regulatory T cells (Tregs) mediate immune tolerance, while Th17 cells are effector cells of the immune-inflammatory response, and a disbalance of either type is associated with inflammatory and autoimmune diseases. Atarashi et al. confirmed that *Clostridium* that had colonized in the gut; particularly the *Clostridium* clusters IV and XIVa; could exert Treg-like induction by increasing TGF-β levels in vivo [42]. This, in turn, promotes Th17 cell generation in vivo and the increased expression of *Foxp3* transcription factors. Recent studies suggest that the nature of the Treg/Th17 imbalance observed in patients with PCOS is inflammatory and involves a significantly increased number of Th17 cells, thereby promoting IR, hyperandrogenemia, and persistent anovulation [43]. However, the molecular mechanisms and related regulatory networks underlying this phenomenon are not completely understood. Normally, levels of CD4^+^ and CD8^+^ cells are counterbalanced with each other in vivo. Correspondingly, too high or too low a CD4^+^/CD8^+^ ratio indicates disordered immunomodulatory function, while peripheral blood with an elevated CD4^+^ lymphocyte count in patients with PCOS has also been shown to be an immune-associated factor underlying PCOS pathogenesis [44].

### The correlation of the gut flora with PCOS inflammatory responses

Chronic inflammation plays an important role in the onset of PCOS and is an important clinical manifestation of the condition. Numerous domestic and foreign studies have found that patients with PCOS have increased levels of serum inflammatory factors, such as C-reactive protein and tumor necrosis factor (TNF-α) [45–47]. LPS in the cell wall component of Gram-negative bacteria can enter systemic circulation via the damaged intestinal mucosal barrier, causing chronic inflammation and endotoxemia in the host [48]. Chronic inflammation caused by a dysregulation of the intestinal flora is closely associated with obesity, IR, and various metabolic diseases. Wang et al. found that the serum levels of LPS and several inflammatory factors (TNF-α, IL-6, and IL-8) were significantly increased in letrozole-induced PCOS rats. In addition, TLR4 expression was evidently elevated in PCOS rat ovaries, which promotes the activation of the NF-κB signaling-mediated inflammatory response in ovarian tissue [49]. Moreover, abnormally elevated serum inflammatory factor levels may induce serine phosphorylation occurring on insulin receptor substrate 1 (IRS 1) in muscles and adipocytes, thereby blocking insulin signaling in peripheral tissues and inducing IR [50] (Fig. 1).

## Polyphenols regulate polycystic ovary syndrome by improving the gut microbiota

### Overview of polyphenols

Phenols comprise a class of plant chemicals, with their highest contents being in plants. They are products of secondary plant metabolism that are produced via the shikimic acid pathway and acetic acid pathway. Plant polyphenols can be divided into two categories based on their chemical structure: flavonoids and nonflavonoids. They exist bound to two main structures: (i) sugar, to form glycosides with strong solubility, or (ii) as free non-sugar-binding substances, namely aglycon [51]. Flavonoids, which are also known as bioflavonoids, comprise a class of plant polyphenols that mainly exist in either a bound or free state in vegetables, fruits, tea, beans, and other foods that are commonly encountered in daily life. Flavones are a subgroup of flavonoids that can be further divided into, isoflavones, flavonols, flavanones, flavanols, arachidoside, and other major subcategories. As polyphenolic compounds, flavonoids exhibit physiological and biochemical functional activities (such as anti-inflammatory, antioxidant, and antiviral activities), owing to their variety of species and unique chemical structures [52, 53]. In addition to flavonoids, phytopolyphenols comprise another type of polyphenol, which encompasses small molecules of phenolic acid (such as caffeic acid, ferulic acid, and chlorogenic acid) and other compounds, such as tannins. Stilbenes and lignans are also polyphenol compounds and found to promote body health via antioxidant, anti-inflammatory and metabolic beneficial ways [54, 55] These nonflavonoid polyphenols also play a role in protecting the intestinal mucosa, optimizing the structure of intestinal flora, and maintaining intestinal health [56–58].

### The correlation between polyphenols and the composition of the gut microbiota

**Fig. 2:**
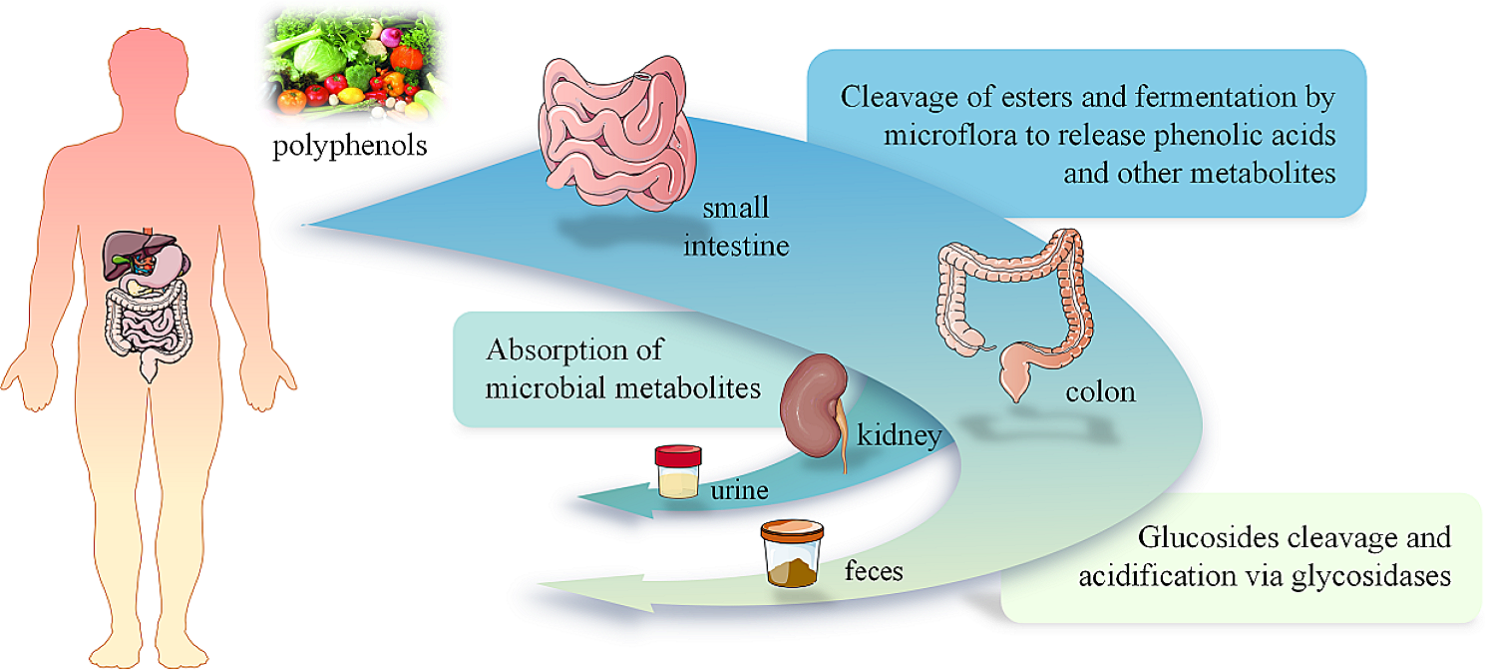
Biotransformation pathway of polyphenols in the human gut

Polyphenols have been shown to play positive roles as anticancer, antibacterial, vasodilation, and analgesic agents by mechanistically producing bioactive, low-molecular-weight metabolites in the human gut. The polyphenol content of different types of berries has been found to reach 200–300 mg/100 g of fresh produce, while one cup of red wine, tea, or coffee contains approximately 100 mg of polyphenols [59]. However, it has also been found that after 50 mg aglycon is absorbed by the human body, its resulting metabolites only reach a concentration of 0–4 μmol/L in the plasma, while the excreted urine contains only 0.3-43% of the intake dose [60]. This finding suggests that despite the high concentrations of polyphenols found present in the diet, they have low bioavailability once consumed. The reason for this is that after their ingestion, polyphenols are recognized as exogenous compounds by the human body, leading to their significantly lower bioavailability compared to other micronutrients and macronutrients. After phenolic substances enter the small intestine, only a small portion of them is absorbed due to their complex structure and polymerization; this absorption mostly occurs after they undergo the decoupling reaction [61]. Another small portion of less-complex phenols may alternatively undergo oxidation, reduction, hydrolysis, and conjugation to subsequently enter small intestinal epithelial cells and hepatocytes. Simultaneously, after phenols undergo a certain degree of biological transformation, multiple active metabolites; such as glucuronic acid, sulfate, and methyl derivatives; are transported via the circulatory system to the tissues and organs, after which, they are excreted with other urine metabolites [61]. However, most polyphenols are not absorbed by the small intestine, instead reaching the colon to become digested and absorbed under the action of microorganisms [62, 63]. The biotransformation path of polyphenols in the human gut is presented in Fig. 2.

Different polyphenols can either promote or inhibit the growth and proliferation of various types of flora. Dietary polyphenols promote the growth of beneficial bacteria and inhibit the growth of harmful bacteria. These abilities affect the structure and quantity of intestinal flora to regulate the stability of human intestinal microecology, thereby allowing beneficial bacteria to play their role in supporting human health. The effects of several dietary polyphenols on the gut flora are listed in Table 1.

**Table 1:** Effects of polyphenols on the composition of the gut microbiota

Polyphenol sources	Polyphenol species	Effects of polyphenols on the gut microbiota	Ref.
Green tea	EGC, C, theophylline, EGCG, etc.	*Bacteroides* abundance increased, whereas *Firmicutes* abundance decreased.	Guo et al. [49]
Grape seed	GA, C, EC	*Bifidobacterium* and *Lactobacillus* contents increased, while *Clostridium histolyticum* and *Prevotella* growth was inhibited, but no significant change in their total number was observed.	Zhou et al. [64]
Wine	EC, EGC, C, anthocyanins, phenolic acids, etc.	*Bifidobacterium* content increased significantly in both *Clostridium* and *Lactobacillus*, but not the total number of bacteria.	Dolara et al. [65]
Red wine; dietary intake	Resveratrol	*Lactobacillus* and *Bifidobacterium* became the dominant genera, alongside relatively decreased levels of *Escherichia coli* and other intestinal bacteria.	Larrosa et al. [66]
Dietary intake	Resveratrol	The proportion of *Bacteroides* and *Firmicutes* increased, while the growth of *Lactobacillus* and *Bifidobacterium* was promoted.	Qiao et al. [67]
Mango	MKE	The growth of Gram-positive bacteria was promoted, thereby suppressing the growth of Gram-negative bacteria.	Kabuki et al. [68]
Plant sources	Propolis	The flora structure was modulated in such a way that relieved inflammatory symptoms.	Wang et al. [69]
Tea	EC, C	The proliferation of *Bifidobacterium* and *Lactobacillus* was promoted, while the microbial abundance of *Firmicutes* (such as *Clostridia*) was reduced.	Tzounis et al. [70]

Abbreviations: epigallo-catechin (EGC); catechin (C); epigallocatechin gallate (EGCG); gallic acid (GA); epicatechin (EC); mango seed kernel extract (MKE)

**Fig. 3:**
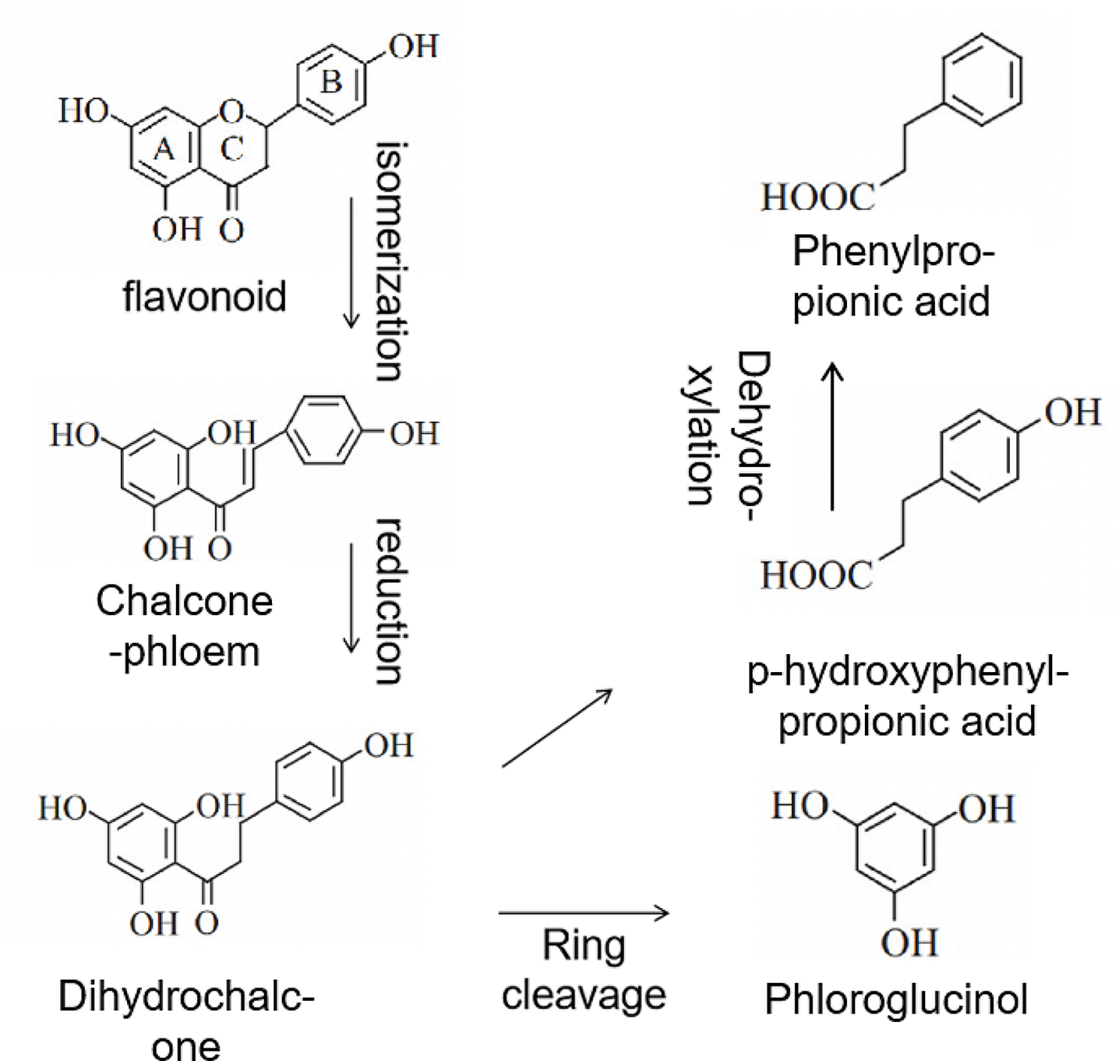
Biotransformation pathway of polyphenols (phloretin) by the gut microflora

The intestinal microbiota can produce different enzymes, such as α-murine plum glycosidase, β-glucuronidase, β-glucosidase, β-galactoglycotase, nitroreductase, nitrogen reductase, 7-α hydroxylase, protease, and various carbohydrate enzymes [71–73]. The metabolic reactions carried out by the intestinal microbiota on polyphenols mainly consist of hydrolysis and reducing reactions. Taking flavonoids as an example, the intestinal flora not only metabolizes flavonoids by O-glycosylation, C-glycosylation, ester hydrolysis, amide hydrolysis, and glucuronidation, but also by dehydroxylation, demethoxy, demethylation, hydrogenation, α-oxidation, β-oxidation, and aromatic ring lysis [74]. The aromatic ring lysis of the flavonside element produces hydroxylated forms of phenylphenol intermediates and either phenylacetate orphenylpropionate. The metabolism of polyphenols by the intestinal microbiota is illustrated in Fig. 3.

Overall, polyphenols can alter the composition of the gut flora, which promotes the growth of beneficial taxa and inhibits that of harmful taxa. Dietary polyphenols from most sources do not inhibit the growth of lactic acid flora, and conversely, may stimulate the growth of certain lactic acid strains [64, 65, 70]. In addition, studies have found that intestinal flora can convert polyphenols into bioactive substances that affect the health of the gut and body. Dietary polyphenols can regulate the enzymatic activities of bacterial metabolites, reduce the risk of cancer, and reduce the risk of inflammatory bowel disease [66, 67].

### Regulatory effect of polyphenols on polycystic ovary syndrome

*Bacteroides* exhibit significantly higher numbers in the gut microbiota of patients with PCOS, which is accompanied by reduced levels of GDCA and TUDCA, both of which stimulate the immune system excessively to cause inflammation [5]. Thus, the role of the gut microbiota in PCOS is gradually becoming better elucidated. Possible treatments for this include interventions that target the diet, microbiome diversity, microbiota-produced toxins, systemic inflammation, the inflammatory response, and lipid metabolism [75]. Among these contributors, dietary intervention is considered the most effective and easily-controlled means to modulate the gut flora [63]. Bioactive compounds derived from foods, such as polyphenols, may serve as promising targets to regulate the gut microbiota. These compounds are powerful antioxidants and natural anti-inflammatory agents that are already widely used to prevent inflammation and oxidative stress in chronic “lifestyle burden” diseases [70]. The results of various studies based on many laboratory models; including in vitro, in vivo, and clinical studies; have demonstrated the positive effects of polyphenols on health [76, 77].

Anthocyanin is an aqueous-soluble phytopigment found extensively throughout various plants, such as grapes and red complex basins. The pigment can improve different health indicators, such as vision, blood pressure, and cognitive ability, and shows utility in preventing heart disease [78]. Recently, anthocyanin was found to improve the dysregulation of ovarian steroids and modulate the expression of steroidogenic enzymes, antioxidant enzymes, and inflammatory markers in androgen-induced PCOS mice, indicating that it exerts a therapeutic effect on PCOS [79]. Furthermore, animal studies have shown that anthocyanins can regulate intestinal activity and alter both the structure and function of the gastrointestinal tract, which may be related to corresponding changes in the composition of the gut microbiota [80–82]. Anthocyanin can promote the growth of *Bifidobacterium*, *Lactobacillus* and *Enterococcus*, all of which actively function to maintain the intestinal mucus, restore the epithelial barrier structure, perform immunomodulation, and regulate the microbiome. In a study conducted by Bibi et al [83], red complex basin anthocyanins were found to exhibit a protective effect on the gut barrier, which was attributed to their ability to significantly inhibit elevated claudin-2 protein levels and increase claudin-3 and ZO-1 expression caused by sodium dextran sulfate treatment. During the onset of PCOS, high concentrations of follicular androgens significantly inhibit the normal development of the ovary. Therefore, serum sex hormone levels should be actively monitored and regulated during the treatment of PCOS. Studies have shown that anthocyanin, which exerts a therapeutic effect on antioxidative stress damage, not only regulates serum sex hormone levels in rats with PCOS, but also improves ovarian morphology [79]. This effect is mediated by the p53/AMPK signaling pathway and controls the transformation of LC3 I to LC3 II, inhibiting autophagy in ovarian granulosa cells [84]. Interestingly, sex steroids are strongly associated with alterations in gut microbiota composition [85]. Choi et al. observed a decreased abundance of Bacteroidetes and an increased abundance of Firmicutes in ovariectomized mice compared to controls [86]. Although the specific mechanism underlying this observation remains unknown, this finding suggests that sex hormones secreted from ovaries modulate gut microbiota composition. Therefore, anthocyanin may ameliorate gut microbiota dysbiosis by reducing serum androgen levels, though this will require experimental confirmation.

Catechins are volatile and prone to degradation and metabolism when reacting with hydroxyl groups on phenolic rings under physiological conditions. Even when administered by intravenous injection, catechins are partly degraded before reaching the target tissue [87]. Catechins are catabolized in the liver, small intestine, and colon [88]. Several recent studies have described the anti-infective properties of primary catechin and epigallocatechin gallate (EGCG) in green tea [89, 90]. The antimicrobial activity of catechins in tea has been extensively investigated and found to be protective against gastrointestinal diseases, such as colon and colon cancer. NF-κB is a nuclear transcription factor that regulates cell proliferation, differentiation, and carcinogenesis. Hong et al. demonstrated that catechins can significantly downregulate uterine p-NF-κB p65 expression and the protein expression of proinflammatory factors (IL-1β, IL-6, and TNF-α), as well as regulate matrix degradation-related MMP2 and MMP9 expression in uterine tissues. These findings suggest that the catechins sourced from oolong tea can inhibit uterine inflammation and matrix degradation by inhibiting p-STAT3 signaling [91]. Additionally, supplementation with green tea extract has been found to significantly reduce serum LH levels in estradiol valerate-induced PCOS rats. Moreover, a reduction in the insulin resistance index was observed in a green tea extract-treated group [92]. Clinical research has shown that supplementation with catechin alleviated abnormal hormone profiles in women with PCOS, which may be attributed to its anti-oxidative effect [93]. However, discarding the fact that the relationship between catechins and the gut microbiota has been extensively explored, whether catechins act by ameliorating gut microbiota dysbiosis to improve PCOS symptoms is still unknown and requires further investigation.

Resveratrol is an antifungal and antibacterial distyrene substance derived from plants. It is found in various fruits, such as grapes (and their juices), oranges, cranberries, currants, and peanut skins [94, 95]. Resveratrol has been demonstrated as an effective antioxidant, antibacterial, anti-obesity, anti-inflammatory, and anticancer agent [96]. Moreover, it is often regarded as an effective scavenger of reactive oxygen species and free radicals. It also exerts a certain protective effect on ovarian function [97–99]. The clinical value of resveratrol in the treatment of PCOS has been confirmed in numerous studies [100, 101]. Research regarding the effect of resveratrol on PCOS has shown it to decrease sinus follicles, increase secondary follicles, reduce granulosa cell death, and reduce oxidative stress levels [100, 102]. Resveratrol has also been shown to reduce androgen levels and increase insulin sensitivity [103]. In a rat model, resveratrol was found to increase the SIRT1 mRNA expression of granulosa cells and deacetylate SIRT1, potentially providing a therapeutic effect on insufficient luteal function [104]. Resveratrol functions in the ovaries by activating not only SIRT1, but also other biological functions that regulate other signaling pathways. Resveratrol exerts its anti-inflammatory and anticancer effects by inhibiting NF-κB activation by TNF-α. The NF-κB/p50 and NF-κB/p65 subunits are present in pig ovarian granulosa cells and SIRT1 can regulate the FSH/NF-κB pathway [105]. Furthermore, Wang et al. reported that resveratrol could protect ovarian follicles from atresia via modulating the SIRT1-FoxO1/P53 pathway in such a way that involved alterations to the gut microbiota. Following microbiota transplantation from donors who consumed a resveratrol-supplemented diet, the Shannon index of recipients was increased, their ratio of Firmicutes/Bacteroidetes was significantly increased at the phylum level, their relative abundances of *Lactobacillus aviarius* and *Lactobacillus salivarius* were increased, and their relative abundance of *Bacillus velezensis* was decreased [98]. These results provide further evidence to indicate the involvement of the gut microbiota in the protective effect of resveratrol on ovarian function, supporting the therapeutic role of resveratrol in ovarian diseases.

Although dietary polyphenols are proved to modulate gut microbiota composition by acting as prebiotics and alleviate the PCOS disease state [106], including insulin resistance and abnormal hormone levels, neither the effect of polyphenols on PCOS gut microbiota, nor the involvement of PCOS-related gut microflora dysbiosis in polyphenols metabolism was thoroughly explored. Commonly found in soy products, isoflavones are found to exert anti-inflammatory and antioxidant effect in multiple diseases [107, 108]. Daidzein, as a kind of isoflavones, is weak ligand of estrogen receptor and exert estrogenic effect. Furthermore, Daidzein can be converted to equol via gut microbiota [109], implying the close relationship between gut microbiota and isoflavones metabolism. It is reported that isoflavones exhibit therapeutic effect on PCOS [110, 111], to explore the effect of isoflavones on PCOS gut microbiome and equol production, Haudam et al. performed 16 S rRNA sequencing of stool samples from PCOS patients after three days of isoflavone intervention and found that isoflavone intervention increased alpha diversity in PCOS group to healthy baseline levels. In addition, the overall prevalence of equol-produced bacteria was 42% (8/19) in control women 21% (5/24) in women with PCOS, which was consistent with the decreased serum equol levels in PCOS patients, suggesting the possible role of isoflavone in modulating PCOS gut microbiota composition. Thus, isoflavone may act as prebiotic and show great potential in PCOS treatment.

## Conclusion

PCOS is a common metabolic and endocrine disorder with a complicated pathophysiology that involves disturbance of gastrointestinal microbiome. The evidence suggests that polyphenols may act as prebiotics, alter microbiome composition, and produce secondary metabolites that modulate host metabolic and ovarian function. Polyphenols and their metabolites also exert the known anti-inflammatory and antioxidant function which significantly alleviate the systemic chronic inflammation in women of PCOS. On the other hand, gut microbiota participated in the metabolism of polyphenols and promoted the absorption of secondary metabolites. Therefore, the regulatory network between gut microbiota and polyphenols in PCOS is complicated. Further studies should be performed to elucidate specific mechanisms by which polyphenols alter the composition of the gut microbiota and the production of secondary metabolites. Knowledge of the metabolic and endocrine effects of dietary polyphenols has the potential to provide novel therapeutic options for women with PCOS.
